# Systems of Risk Stratification of Malignancy by Ultrasound of Thyroid Nodules

**DOI:** 10.7759/cureus.11424

**Published:** 2020-11-10

**Authors:** Luis Antonio Rodriguez Arrieta, Alejandro Roman-Gonzalez, Carlos A Builes Barrera

**Affiliations:** 1 Endocrinology, University of Antioquia, Medellin, COL; 2 Endocrinology, University of Antioquia, Hospital San Vicente Foundation, Medellin, COL

**Keywords:** thyroid nodule, ultrasonography, thyroid cancer

## Abstract

Thyroid nodules (TN) are more frequently identified with the use of thyroid ultrasonography, and they have a low risk of malignancy. Ultrasonographic features have been established that increase the probability of being faced with thyroid carcinoma; however, individually, these characteristics do not perform adequately in the diagnosis of malignancy, limiting their usefulness when indicating cytological studies by means of fine-needle aspiration (FNC). This situation motivated the development of risk stratification systems for thyroid nodules, which unified their ultrasound characteristics, with the aim of establishing risk categories, standardizing the preparation of reports, and providing the clinician with useful tools to define the surveillance option or form invasive studies.

The objective of this review is to compare the different systems developed by some scientific societies for the stratification of thyroid nodules, with respect to their predictive capacities for malignancy, their operational characteristics for diagnosis, and, to suggest recommendations for the implementation of these systems, placing emphasis on those with the best ability to reduce the performance of unnecessary invasive studies and to guide decision-making in the face of undetermined cytological results.

## Introduction and background

Thyroid nodules (TN) are common, found through palpation in 4%-7% and through thyroid ultrasound (US) in up to 70% of the population [1]. Actually, fine-needle aspiration biopsy (FNA) is the main tool to differentiate a benign from a malignant TN. Most TN (>90%) are not cancer; others are small and inert malignancies; therefore, FNA has to be restricted for those TN with clinical or radiological characteristics associated with thyroid carcinoma (TC) suspicion. Low-risk TN can undergo follow-up without any invasive procedure, avoiding complications and additional costs [1-4].

US suspicious findings are well-established, even though the report is operator-dependent [5]. Given the imaging findings, the clinician must establish which nodule to work up and which to follow up [5-6]. Hence, scientific societies recommend ultrasound risk stratification systems (RSS) to standardize reports and to make easier clinical decision-making [1-4] (to do or not FNA and further follow-up). The objective of this review is to compare the different RSS on their malignancy predictive value, diagnostic performance, and, additionally, to suggest recommendations to implement such systems, with an emphasis on those systems that may diminish the number of unnecessary invasive tests and guide in clinical decision-making when an undetermined cytologic result is observed. A narrative review was carried out using the Medline, Pubmed, Elsevier, Embase, and Google Scholar search engines, and the bibliographic documents published up to April 2020 were located. Words such as thyroid nodule, risk stratification systems, thyroid carcinoma, and thyroid ultrasound were used.

## Review

Ultrasound risk stratification systems

Thyroid ultrasound is the first-line study to evaluate the risk of malignancy of a TN. It has been proven that some US characteristics increase the risk of malignancy, such as hypoechoic findings in the solid part of a nodule, taller-than-wide, irregular or spiculated margins with absent halo, microcalcifications, and signs of extra-thyroidal growth. The malignancy risk associated with each characteristic is substantial, but none of these findings has a high enough diagnostic performance (sensitivity and specificity) to be used isolated one of the other [7]. Furthermore, these characteristics are subject to interpretation and affected by external factors such as the ultrasound equipment, the configuration and resolution of the screen, and the operator’s criteria. As a result, the interobserver and intraobserver concordance for each individual characteristic is moderate (Cohen’s Kappa coefficient: 0.4-0.6) [7].

Since 2005, multiple attempts have been done to diminish such limitations with US RSS [8]. These systems look to reduce the overdiagnosis and overtreatment of the current incidental TN “epidemic” [8]. RSS may differ one from the other. The US characteristics included in the RSS by the British Thyroid Association (BTA) [5], American Thyroid Association (ATA) [3], American Association of Clinical Endocrinologists (AACE), and Associazione Medici Endocrinologi (AME) [1] are shown in Table 1. These three systems categorize a TN based on the echographic pattern associated with a higher or lower risk of malignancy. Nonetheless, since the 2009 proposal of Horvath/Thyroid Imaging Reporting and Data System (TIRADS-Horvath) and further versions developed by the KWAK Thyroid Imaging Reporting and Data System (KWAK-TIRADS), Korean Thyroid Association (KTA)/Korean Society of Thyroid Radiology (KSThR), American College of Radiology-Thyroid Imaging Reporting and Data System (ACR-TIRADS), and the European Thyroid Association (ETA), a stratification score is used to determine the possibility of a TC [2,4-6,8].

**Table 1:** Benign and malignant US characteristics included in different RSS Performed by the authors based on different cited guidelines in this review. TIRADS: Thyroid Imaging Reporting and Data System; BTA: British Thyroid Association; ATA: American Thyroid Association; AACE: American Association of Clinical Endocrinologists; ACE: American College of Endocrinology; AME: Associazione Medici Endocrinologi; KSThR: Korean Society of Thyroid Radiology; KTA: Korean Thyroid Association; ACR: American College of Radiology; ETA: European Thyroid Association

Ultrasound (US) characteristics	Horvath-TIRADS (2009)	Kwak-TIRADS (2009)	BTA (2014)	Revised ATA (2015)	AACE/ACE/ AME (2016)	KSThR/ KTA (2016)	ACR TI-RADS (2017)	ETA (2017)
Benign US characteristics								
Cystic lesions	X	X	X	X	X	X	X	X
Spongiform/honeycomb pattern	X	X	X	X		X	X	X
Mostly cystic nodules with liquid component and no suspicious signs associated	X	X	X	X	X	X	X	
Mostly cystic nodules with an eccentric solid pole	X	X	X	X	X	X	X	
Isoechoic/hyperechoic nodule	X	X	X	X		X	X	X
Isoechoic/hyperechoic nodule with halo			X			X		
Oval shape						X		
Comet tail artifact		X	X			X	X	X
Peripheral egg-shell like calcification			X			X		
Regular margins		X	X	X		X	X	
Absent vascularization						X		
Peripheral vascularization	X		X	X		X		
Regular halo			X			X		
Malignant US characteristics								
Hypoechoic solid component	X	X	X	X	X	X	X	X
Markedly hypoechoic	X	X			X	X	X	X
Irregular shape	X	X	X	X	X	X	X	X
Microcalcifications/punctate echogenic foci	X	X	X	X	X	X	X	X
Calcifications Macrocalcificaciones /globular calcifications	X	X	X		X	X	X	
Disrupted peripheral rim calcifications	X			X	X			
Incomplete or broken peripheral calcification	X	X			X	X	X	
Hyperechoic spots of undetermined significance					X			
Irregular or spiculated margins with absent halo	X	X		X	X	X	X	X
Intranodular vascularization		X	X	X	X	X	X	
Mixed vascularization (central and peripheral)								
Invasion of thyroid parenchyma	X		X		X	X	X	
Subcapsular or paratracheal lesions					X			
Pathologic adenopathy (hilum absence, microcalcifications, cystic degeneration, spherical shape, diffuse or peripheral hyper vascularization, hyperechoic aspect)			X	X	X			
Central adenopathy with a minor axis >8 mm. Lateral adenopathy >10 mm			X	X				
Metastasis			X	X				

RSS has different categories, each with an estimated malignancy probability. These percentages were initially determined by expert consensus; the inter-system variability limits direct comparison as shown in Table 2. Some systems are derived from population data used in previous systems, without a proper validation study [9]. Regardless, in the last years, prospective validation in different cohorts has been performed.

**Table 2:** Risk stratification systems and their categories. Performed by the authors based on different cited guidelines in this review. TIRADS: Thyroid Imaging Reporting and Data System; BTA: British Thyroid Association; ATA: American Thyroid Association; AACE: American Association of Clinical Endocrinologists; ACE: American College of Endocrinology; AME: Associazione Medici Endocrinologi; KSThR: Korean Society of Thyroid Radiology; KTA: Korean Thyroid Association; ACR: American College of Radiology; ETA: European Thyroid Association

RSS	Category and estimated malignancy probability				
ATA (2015)	Benign (<1%)	Very low-suspicion (<3%)	Low suspicion (5-10%)	Intermediate suspicion (10-20%)	High suspicion (70-90%)
BTA (2014)	U1-Normal (0%)	U2-Benign (<1%)	U3-Indeterminate (3-10%)	U4-Suspicious (10-20%)	U5- Malignant (70-90%)
AACE/ACE/AME (2016)	Low-risk (1%)		Intermediate-risk (5-15%)	High-risk (50-90%)	
KSThR/KTA (2016)	K-TIRADS 1 (0%)	K-TIRADS 2 (0%)	K-TIRADS 3 (7.8%)	K-TIRADS 4 (25.4%)	K-TIRADS 5 (79.3%)
ACR TIRADS (2017)	TR1 - Benign (0%)	TR2 – Not suspicious (0%)	TR3 – Mildly suspicious (<5%)	TR4 - Moderately suspicious (5-80%) TR4a: 5-10% TR4b: 10-50% TR4b: 50-80%	TR5 -Suspicious (>80%)
ETA (2017)	EU-TIRADS 1 No nodule (0%)	EU-TIRADS 2 Benign (0%)	EU-TIRADS 3 Low risk (2-4%)	EU-TIRADS 4 Intermediate risk (6-17%)	EU-TIRADS 5 High-risk (26-87%)

Although these RSS are referenced in different guidelines, multiple difficulties may arise for daily use, whether it is for the technical conditions or physician work-load that may limit the duration of the US study. This may lead to sub-reports that do not meet the minimal characteristics, forcing them to perform another US evaluation to obtain the proper report.

Diagnostic performance of risk stratification systems

Given the rising use of RSS in clinical practice, multiple questions arise about its true diagnostic performance and the strengths and weaknesses of each system [8].

It is important to embrace guidelines to evaluate a diagnostic test from a critical perspective [10]. The first condition to evaluate a diagnostic test is the availability of a gold standard: the new test must offer at least the same diagnostic performance as the reference test. In the case of RSS, the underlying evidence has derived mainly from the population with papillary thyroid cancer (PTC). This introduces an important selection bias because an FNA can adequately diagnose a PTC, but this isn’t the case for follicular carcinomas (FTCs) or medullary carcinomas (MTCs). FTC are indistinguishable from follicular adenomas and usually end up in an undetermined category [8].

MTC are misdiagnosed by FNA in up to 50% of cases [7]. However, there are multiple manuscripts that evaluated the diagnostic performance of RSS as compared to pathology reports of excisional biopsies (gold standard) of high/intermediate risk that underwent surgery, concluding a superior diagnostic performance than FNA. None of the RSS has a 100% capacity to determine the presence of TC. Nonetheless, RSS overall has a higher diagnostic performance and reproducibility than each of the individual risk characteristics [7-9].

In most RSS, an FNA indication is based on the TN US pattern and size. Each RSS establishes a different size limit to perform FNA in each risk category. Shen et al. retrospectively evaluated the US digital images of 1568 patients (1612 TN) that underwent surgery in a reference center. All the TN were histologically classified as benign or malignant. In Table 3, the diagnostic performance of four RSS is described according to the size of the TN in the study performed by Shen et al. [9]. Given the retrospective nature of the data, the validity of such findings may be compromised.

**Table 3:** Diagnostic performance of four RSS for TN of different sizes. RSS: risk stratification system; SEN: sensitivity; ESP: specificity; PPV: positive predictive value; NPV: negative predictive value; AUC: área under the curve; ATA: American Thyroid Association; ACR: American College of Radiology; ETA: European Thyroid Association; K-TIRADS: KWAK-Thyroid Imaging Reporting and Data System Performed by the authors based on different cited guidelines in this review.

Size	RSS	SEN (%)	SPE (%)	PPV (5)	NPV (%)	AUC (%)
< 10 mm	ATA ACR ETA K-TIRADS	96.4 95.3 97.7 97.4	85.1 66.5 61.1 64.7	85.1 86.7 85.2 86.2	87.9 86.1 91.9 91.5	78.6 (74.0-83.3) 81.1 (76.7-85.6) 79.4 (74.7-84.0) 80.8 (76.3-85.4)
>10 y < 20 mm	ATA ACR ETA K-TIRADS	86.8 81.9 88.0 88.4	81.5 88.0 80.4 86.2	77.4 83.3 76.6 82.4	89.4 87.0 90.1 91.0	84.4 (81.0-87.9) 85.1 (81.6-88.6) 84.2 (80.6-88.6) 87.3 (84.1-90.5)
> 20 mm	ATA ACR ETA K-TIRADS	88.0 79.7 91.0 91.7	93.0 97.6 91.8 96.3	83.6 93 81.8 91	91.5 92.4 97.4 96.0	90.5(86.9-94.2) 88.6 (84.6-93.3) 91.4 (88.1-94.9) 93.9 (91.1-97.2)

Zhang et al. prospectively categorized 3980 TN (3752 benign lesions and 228 malignant) between October 2011 and June 2013 in 2921 outpatients in Shanghai, China, using the KWAK-TIRADS system. The sensitivity, specificity, positive predictive value (PPV), negative predictive value (NPV), and precision were 97%, 90%, 40%, 99%, and 91%, respectively [11]. K-TIRADS has been validated by a prospective, multicentric study of four institutions with 902 TN. Sensitivity, specificity, PPV, NPV and precision for malignant TN were 95.5%, 58.6%, 44.5%, 96.9% and 69.5%, respectively [12]. The prospective validation of EU-TIRADS showed a 86.1% sensitivity, 32% specificity, 8.9% PPV, and 96.7% NPV [13].

These data were extracted from individual prospective validation studies; unfortunately, these results are not enough to establish the best system [11-13].

Recently, in a prospective study with 987 TN sent for FNA, the diagnostic accuracy for the malignancy of the RSS proposed by BTA, ATA, and AACE/ACE/AME was evaluated, comparing the high risk to the low/intermediate-risk categories and high/intermediate-risk to low-risk categories (Table 4) [14]. The conclusion is that the three systems have a similar diagnostic performance. The reported negative predictive value (NPV) is equal or higher to 95%, a useful aspect to evaluate TN, especially those in the low-risk category, as those TN have a low pre-test probability for malignancy, follow-up can be performed by US alone [14].

**Table 4:** Diagnostic performance for the malignancy of the BTA, ATA, and AACE/ACE/AME risk stratification systems, comparing low and intermediate risk vs high risk RSS: risk stratification system; SEN: sensitivity; ESP: specificity; PPV: positive predictive value; NPV: negative predictive value; TIRADS: Thyroid Imaging Reporting and Data System; BTA: British Thyroid Association; ATA: American Thyroid Association; AACE: American Association of Clinical Endocrinologists; ACE: American College of Endocrinology; AME: Associazione Medici Endocrinologi Performed by the authors based on different cited guidelines in this review.

RSS	BTA	ATA	AACE/ACE/AME
Risk category	U2 and U3 vs U4 and U5	1,2,3 and 4 vs 5	1 and 2 vs 3
SEN	74%	81%	82%
SPE	92%	87%	87%
PPV	62%	54%	54%
NPV	95%	96%	96%
Precision	89%	86%	86%
Risk category	U2 vs U3, U4, and U5	1 and 2 vs 3, 4, and 5	1 vs 2 and 3
SEN	90%	98%	99%
SPE	63%	21%	21%
PPV	32%	19%	19%
NPV	97%	98%	99%
Precision	68%	33%	34%

Despite the researchers' efforts, no RSS has been widely embraced, and there are some contradicting recommendations between different institutions [8,14].

Strategies to reduce unnecessary thyroid biopsies

The target of the TIRADS system is to improve clinical decision-making and to reduce unnecessary FNA (7). The rate of unnecessary FNA to diagnose TC was defined as the percentage of benign TN divided by the total number of patients that underwent FNA. Xu et al. observed an unnecessary FNA rate of 25.2%, based on EU-TI-RADS [15].

Recently, Castellana et al. included 12 studies in a meta-analysis with 18750 TN. The population included was outpatient adults with TN that underwent FNA, core needle biopsy, or surgery that had US images available. The final diagnosis of malignant nodules was based on histology; cytology was used for diagnosis in benign nodules. The diagnostic odds ratio (DOR) was 2.2-4.9. A head-to-head comparison to select TN that should undergo FNA observed a higher DOR for ACR-TI-RADS against ATA (p = 0.002) or K-TI-RADS (p = 0.002); hence, ACR-TI-RADS yielded a higher diagnostic performance to select patients that had to undergo an invasive procedure. Nonetheless, these results must be confirmed in further studies [16].

In another study by Grani et al., 902 TN were evaluated, and the rate of unnecessary FNA was lower with ACR-TIRADS (25.8%), followed by ATA (51.2%), and K-TI-RADS (59.4%) [6]. The ACR-TI-RADS system was superior to the other systems, identifying more than 50% of biopsies as unnecessary, and with a lower false negatives rate. This difference is due to the size threshold for FNA in each RSS, as shown in Table 5 [6].

**Table 5:** Size threshold for FNA indication in five different RSS FNA: Fine-needle aspiration biopsy; TIRADS: Thyroid Imaging Reporting and Data System; ATA: American Thyroid Association; AACE: American Association of Clinical Endocrinologists; ACE: American College of Endocrinology; AME: Associazione Medici Endocrinologi; KSThR: Korean Society of Thyroid Radiology; KTA: Korean Thyroid Association; ACR: American College of Radiology; ETA: European Thyroid Association Performed by the authors based on different cited guidelines in this review.

Size threshold for FNA indication in each system				
AACE/ACE/AME (2016)	ACR TIRADS (2017)	ATA (2015)	KSThR/ KTA (2016)	ETA (2017)
High-risk FNA >10 mm	TR5 FNA > 10 mm	High-risk FNA > 10 mm	TR5 FNA > 10 mm	TR5 FNA > 10 mm
Intermediate-risk FNA >20 mm	TR4 FNA > 15 mm	Intermediate-risk FNA > 10 mm	TR4 FNA > 10 mm	TR4 FNA > 15 mm
	TR3 FNA > 25 mm	Low-risk FNA > 15 mm	TR3 FNA > 15 mm	TR3 FNA > 20 mm
Low-risk FNA >20 mm	TR2 No FNA	Very low-risk FNA > 20 mm	TR2 FNA > 20 mm	TR2 No FNA
	TR1 No FNA	Benign no FNA	-	-

In conclusion, to reduce the number of unnecessary biopsies, FNA is recommended for TN of intermediate risk (TIRADS category 3) with a size > 25 mm, and TIRADS category 4 with a size > 15 mm [2,17].

The role of clinical examination, ultrasound, and new biopsy in the follow-up of benign TN is unclear. In most cases, clinical examination, ultrasound, and thyroid-stimulating hormone are performed at six to 18 months in patients in whom biopsy is not indicated. A new FNA can be considered if a TN increases in size, defined as an increase of at least 20% in two dimensions (minimum 2 mm), if cystic degeneration is observed, or in case of suspect clinical or ultrasound changes [2-4].

Can risk stratification systems rule out malignancy in cytologically undetermined thyroid nodules?

The Bethesda system for reporting thyroid cytopathology classifies FNA cytology into six categories [17]. For both benign and malignant TN, further management is quite clear [1-5]. However, undetermined results can portray a challenge for clinical decision-making: to repeat or not to repeat FNA, perform thyroid surgery, or follow-up. This challenge is due to most TN with undetermined FNA results (atypia of undetermined significance AUS/follicular lesion of undetermined significance (FLUS), Follicular neoplasm, and suspicious for malignancy) are histologically benign, with a malignancy risk of 10-30% for Bethesda III (B-III) and 25%-40% for B-IV [18].

The malignancy uncertainty of undetermined samples leads to uncertain treatment selection [5,18]. Some studies reported that a repeat FNA might obtain a definitive diagnosis in the B-III category; however, 10%-30% nodules keep the same undetermined results, hence making a case against repeat FNA [17]. In those cases where two FNAs had undetermined results and surgery was performed with benign surgical pathology results, the definitive pathology result suggested that the surgical approach was unnecessary [9,19]. These cases may benefit from molecular tests to improve diagnostic precision. However, molecular tests are expensive, which can lead to diagnostic delays and routine clinical use limitations [1-4].

In the last few years, alternative, less invasive approaches have been proposed to reduce unnecessary interventions, costs, and complications [18,20].

When a TN has a B-III cytology, with a TIRADS 2, 3. and 4a US report (estimated malignancy risk of 0-10%), a repeat FNA at six to 12 weeks may cause “changes due to external factors,” leading to degenerative processes in the TN through a hemorrhage, granulation tissue, fibrosis, and sclerosis [5,9 20]. These changes can be observed as spontaneous degenerative TN changes, called “changes due to internal factors," which can lead to confusion in cytological evaluation [21-22].

In subjects with undetermined cytology (especially in the B-III and B-IV categories), a correlation between RSS with cytological category has been proposed, to further define on whom to perform follow-up, molecular tests, or surgery. In a retrospective study with 140 definitive pathology, US, and FNA results, a correlation between low-risk US categories (according to ATA and ACR-TIRADS) and low risk for malignancy in cases with a B-III cytology [23]. Barbosa et al. reported that in cases with B-III cytology that also had a TIRADS category 2, 3, and/or 4, or a very low-risk/low risk/intermediate ATA category, the NPV to rule out malignancy was 94.1% and 94.3%, respectively, favoring a conservative approach in this scenario. Nonetheless, in cases with B-III cytology and higher US risk categories (TI-RADS 4b, 4c, and 5; high-risk ATA), the NPV was 68 and 45,5%. The malignancy risk is higher in these cases, and it isn’t possible to rule out malignancy with US alone [23].

A pragmatic approach to undetermined TN, published in 2017, proposed the following clinical decision-making: in TN with a B-III result, the US must be re-visited. If high-risk characteristics are observed (TIRADS: 4b, 4c, and 5), the surgical approach is recommended [23]. In TN with a B-IV result, RSS utility is limited to rule out malignancy and must not be used in this scenario for clinical decision-making. If there are no molecular tests available, surgery can be considered [24].

## Conclusions

Ultrasound RSS are tools that must be used in the TN workup. These systems have more similarities than differences. The clinical decision-making suggestions in this review are done to reduce unnecessary invasive procedures and complications and decrease associated healthcare costs. Among the results of prospective studies and a recent meta-analysis, ACR-TIRADS has shown higher diagnostic performance as compared to ATA, BTA, EU-TIRADS, and K-TIRADS; it has also shown better classification to either perform FNA or continue follow-up in low-risk TN.

For the development of this narrative review, the clinical practice guidelines published by the different international scientific societies were included in addition to the original research works that evaluate the usefulness and performance of RSS. A limitation evidenced in these publications was the retrospective nature of an important number of studies, so we gave priority to those with a prospective methodological design to reduce the possibility of bias. The data described is consistent and allows us to conclude that the use of a validated RSS, such as ACR-TIRADS, must be embraced, to standardize the same language, and to obtain consensus in challenging clinical situations as those previously exposed, where molecular tests or a repeat FNA may be less cost-effective than a well-performed ultrasound evaluation.
